# Comorbidities and treatment of somatoform disorders before switching to DSM-5 and ICD-11: what to consider

**DOI:** 10.1192/j.eurpsy.2022.496

**Published:** 2022-09-01

**Authors:** R. Marinescu, M. Fadgyas Stanculete

**Affiliations:** 1 Iuliu Hatieganu University of Medicine and Pharmacy Cluj-Napoca, Department Of Neurosciences - Psychiatry, Cluj-Napoca, Romania; 2 Cluj County Emergency Hospital, 2nd Psychiatric Clinic, Cluj-Napoca, Romania

**Keywords:** somatoform, comorbidities, Treatment

## Abstract

**Introduction:**

Somatoform disorders, previously diagnosed according to DSM-IV-TR and ICD-10, are shifting towards somatic symptom disorder in DSM-V and bodily distress disorder in ICD-11.

**Objectives:**

Before using the current criteria, because the new diagnostic entities can identify a larger pool of patients with various physical complaints and diagnoses, it is essential to consider the physical and psychiatric comorbidities that have an important role in deciding the pharmacological treatment.

**Methods:**

We conducted a retrospective observational study on a group of 169 patients previously diagnosed with a type of somatoform disorder and hospitalized between January 2015 - January 2021 in a psychiatric emergency hospital in Cluj-Napoca, Romania.

**Results:**

Male:female ratio was 1:1.41. The mean age was 52.35±13.3 years, the mean period of hospitalization was 12±5.39 days. 54% of patients lived in urban areas, and almost half of them were married. Most patients were not professionally active and did not receive a superior education. Most patients had one hospitalization and had at least one physical and one psychiatric comorbidity. The most frequent somatic comorbidities were: cardiovascular, metabolic, rheumatological, gastrointestinal, endocrinological, and neurological, and the most frequent psychiatric ones were: depressive, personality, anxiety, neurocognitive, and substance use disorders. The most frequent type of somatoform disorders were: undifferentiated somatoform disorder and somatization disorder. Regarding psychiatric treatment, antidepressants, antipsychotics, benzodiazepines, anticonvulsants, and hypnotics were used. No correlations were observed between the presence of depressive or anxiety disorders and somatic comorbidities.

**Conclusions:**

ICD and DSM need to clarify diagnostic criteria and develop therapeutical guidelines for this type of patient.

**Disclosure:**

No significant relationships.

